# Limited Influence of Oxygen on the Evolution of Chemical Diversity in Metabolic Networks

**DOI:** 10.3390/metabo3040979

**Published:** 2013-10-16

**Authors:** Kazuhiro Takemoto, Ikumi Yoshitake

**Affiliations:** Department of Bioscience and Bioinformatics, Kyushu Institute of Technology, Kawazu 680-4, Iizuka, Fukuoka 820-8502, Japan

**Keywords:** metabolism, chemoinformatics, environmental interaction, evolution

## Abstract

Oxygen is thought to promote species and biomolecule diversity. Previous studies have suggested that oxygen expands metabolic networks by acquiring metabolites with different chemical properties (higher hydrophobicity, for example). However, such conclusions are typically based on biased evaluation, and are therefore non-conclusive. Thus, we re-investigated the effect of oxygen on metabolic evolution using a phylogenetic comparative method and metadata analysis to reduce the bias as much as possible. Notably, we found no difference in metabolic network expansion between aerobes and anaerobes when evaluating phylogenetic relationships. Furthermore, we showed that previous studies have overestimated or underestimated the degrees of differences in the chemical properties (e.g., hydrophobicity) between oxic and anoxic metabolites in metabolic networks of unicellular organisms; however, such overestimation was not observed when considering the metabolic networks of multicellular organisms. These findings indicate that the contribution of oxygen to increased chemical diversity in metabolic networks is lower than previously thought; rather, phylogenetic signals and cell-cell communication result in increased chemical diversity. However, this conclusion does not contradict the effect of oxygen on metabolic evolution; instead, it provides a deeper understanding of how oxygen contributes to metabolic evolution despite several limitations in data analysis methods.

## 1. Introduction

Metabolic networks [1,2,3] can be defined as a series of biochemical reactions that are essential for physiological functions and are responsible for maintaining life. As a result, it is important and interesting not only for researchers in the field of basic biology but also in biotechnology and medical research to understand these networks. In particular, how metabolic networks have evolved in response to changing environments in evolutionary history is a long-standing question in biology.

Recently, several new technologies and high-throughput methods have generated a large amount of genomic and metabolic network data. As a result, investigators have been actively carrying out comprehensive data analyses in an attempt to shed light on the germination and connections of metabolic networks (reviewed in [4,5], for example). In particular, several studies have discussed possible mechanisms underlying the evolution of metabolic networks and their contributions to environmental adaptation (reviewed in [6,7,8]).

In examining evolution, the contribution of oxygen is an interesting example. Specifically, the emergence of oxygen on Earth is thought to have promoted species diversity and morphological complexity [9,10,11,12] (e.g., in the Cambrian explosion of species diversity). The occurrence of atmospheric oxygen during the Devonian period is associated with radiation of terrestrial plants and large predatory fish [13]. As a result, animal body size increased over evolutionary history [14].

Macroscopic evolution is the result of the accumulation of microscopic or molecular changes. Recent studies have reported the mechanism by which oxygen accelerates biological evolution from a molecular viewpoint. For example, a molecular clock for protein folds revealed an increase in the number of protein families due to the emergence of oxygen, as well as the origin and history of aerobic metabolism [15,16].

In this context, the effect of oxygen on metabolic evolution has been examined. In particular, Raymond and Segré [17] identified oxic reactions, which occur in the presence of oxygen, using the concept of scope [18], a computational framework used to characterize the biosynthetic capability of a network when it is provided with certain external resources. They found that oxic reactions occur largely at the periphery of metabolic networks. Moreover, they showed that the available space of metabolic networks in strict and facultative aerobes was more expanded than that in anaerobes. These results suggest that oxygen triggers metabolic network expansion.

Moreover, further chemoinformatic investigation [19] revealed the effect of oxygen on metabolic evolution by comparing the chemical properties of metabolites of oxic reactions and anoxic reactions. In particular, oxic reactions lead to the development of diverse chemical structures. This result is consistent with the expansion of metabolic networks due to oxygen availability, as described above. Furthermore, many oxic metabolites, which are synthesized through oxic reactions, are related to environmental adaptations such as defense against biotic factors and protection of organisms against oxidation. In addition, oxic metabolites appear to be more hydrophobic and rigid than anoxic metabolites. This tendency is consistent with the fact that membranes function to serve such environmental adaptations.

Thus, oxygen promotes the chemical diversity of metabolic networks. This conclusion corresponds to oxygen-driven evolution, a widely accepted hypothesis in biology. However, this hypothesis can be questioned for two reasons.

A previous study [17] did not consider phylogenetic relationships when evaluating the association between network expansion and oxygen requirements, although multiple species belonging to different domains were compared. Previous studies [20,21,22,23,24,25] have reported that metabolic network structures differ between species domains, lifestyle (e.g., free-living or symbiotic), and growth conditions (e.g., growth temperature). Such species traits may be taxonomically constrained; thus, phylogenetic comparative methods [26,27,28,29] are required to control for phylogenetic relationships among species in a correlation study between species traits. Rezende [30] also pointed out the necessity of phylogenetic comparative analysis when discussing the contribution of oxygen to evolution.

Second, a comparison of the chemical properties [19] of oxic metabolites and anoxic metabolites was performed using the integral metabolic network (*i.e*., the reference pathways) obtained by summarizing the metabolic networks of all species, which are available in databases. Discriminative oxic metabolites are reported to be secondary metabolites and hormones [19]. However, these metabolites are expected to appear to be characteristically observed in higher organisms, suggesting strongly biased evaluation. In particular, it remains possible that differences in chemical properties between oxic metabolites and anoxic metabolites result from comparing metabolites characteristically observed in higher organisms and microbes rather than comparing aerobe-specific metabolites and anaerobe-specific metabolites. To reduce the effect of sampling bias, the distribution of the degree of chemical difference between oxic metabolites and anoxic metabolites in each metabolic network of various species must be evaluated using metadata (e.g., oxygen requirement and multicellularity) for specific species.

In this study, we evaluated the association between oxygen availability and metabolic evolution using a phylogenetic comparative method and metadata analysis. We discuss the effect of oxygen on chemical diversity in metabolic networks.

## 2. Results and Discussion.

### 2.1. Metabolic Network Expansion Does Not Conclude When Taking Into Account Phylogenetic Relationships

Raymond and Segré [17] investigated the increased number of metabolic reactions (or enzymes) in 44 organisms after including oxygen, and used known oxic reactions and anoxic reactions as a parameter. They found that network expansion due to oxygen use was highest in eukaryotes and aerobic prokaryotes. This result supports the hypothesis that oxygen contributes to metabolic network expansion.

We re-evaluated this hypothesis using a larger dataset of metabolic networks, compared with the previous study [17]. In particular we focused on 174 organisms (see Section 3.1 and Table S1 in supplementary). This dataset covers most (~90%) of 44 organisms, which were investigated in the previous study, indicating that it includes ~4 times as many organisms as, compared with the dataset in the previous study. Furthermore, 174 organisms in our dataset can be classified into 105 genera, which have ~2.5 times the number of (44) genera, investigated in the previous study. Although phylogenetic heterogeneity in our dataset is slightly higher than that in the previous study, we concluded that a more comprehensive analysis is possible using this phylogenetic tree. In addition, this difference in phylogenetic heterogeneity did not pose a significant problem in this study because we obtained a similar conclusion using our dataset when performing traditional statistical tests, as in the previous study. In particular, we investigated the increase in enzyme and metabolite development. As in the previous study, the increased rate in oxic *versus* anoxic network size was calculated based on oxic and anoxic reactions (see Section 3.3), and was defined as the ratio of the total number of enzymes (metabolites) to anoxic enzymes (metabolites) (*i.e*., metabolic space, which can be available without oxygen).

**Figure 1:**
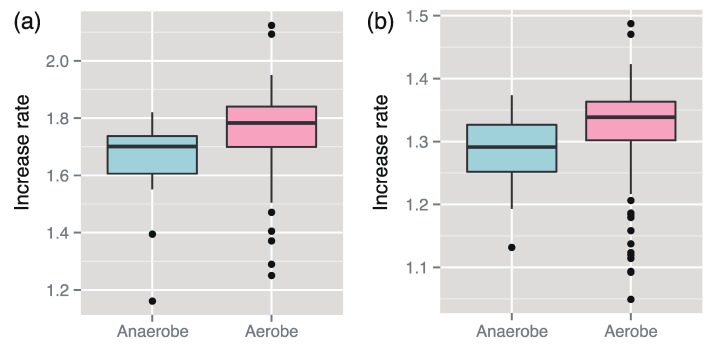
Median comparison of the increase rates between aerobes and anaerobes. The medians can be concluded to be significantly different between them in both case of the enzyme-based increase rates (**a**) (*p*-value *p =* 4.6 × 10^−5^ using the Wilcoxon–Mann–Whitney (WMW) test) and the metabolite-based increase rate (**b**) (*p =* 5.2 × 10^−5^ using the WMW test).

We found that the increased rates of 145 aerobes (including facultative aerobes) were larger than those of 29 anaerobes (Figure 1). A similar result could also be derived through statistical analysis using the linear model (Table 1b), which was performed using the lm function in a statistical software R version 3.0.0 [31].

**Table 1:** Statistical tests for the impact of species oxygen requirements on the increase rate, calculated based on the number of enzymes and the number of metabolites, using linear models with **(a)** and without **(b)** consideration of a phylogenetic relationship. Estimates are shown for the categorical variable corresponding to species oxygen requirements (*i.e*., aerobic or not aerobic). In particular, in this study, we considered that the *p*-value of less than 0.01 indicates a non-zero estimate. A positive estimate means that the increase rate of aerobes is higher than that of anaerobes.

Phylogeny	Enzyme context			Metabolite context		
	Estimate ± SE	*t*-value	*p*-value	Estimate ± SE	*t*-value	*p*-value
(a) Considered	0.04 ± 0.05	0.74	0.47	0.02 ± 0.02	0.83	0.42
(b) Not considered	0.10 ± 0.03	3.8	2.2 × 10^−4^	0.04 ± 0.01	3.0	3.5 × 10^−3^

Statistical analyses indicated that oxygen expands metabolic networks, as reported in a previous study; however, more careful examination is required as traditional statistical methods assume independence of all observations (or star phylogeny; see [26,29] for details). In particular, this traditional statistical method assumes that anaerobes are ancestral. As explained in Section 1, such an assumption may be not satisfied because species traits such as increased rates in metabolic networks and oxygen requirements depend upon the phylogenetic relationship (*i.e*., evolutionary history). In fact, anaerobes are discretely distributed in the phylogenetic tree (Figure S1 in supplementary), suggesting that it remains possible that an evolutionary history (*i.e*., gain and loss of oxygen requirements) causes the observed association between the network expansion and oxygen requirements. To control for phylogenetic relationships among species, therefore, we performed phylogenetic comparative analysis, which can test an association between traits with consideration of such an evolutionary history (see also Section 3.1), using the brunch algorithm [28], which calculates phylogenetically independent contrasts for linear models including binary categorical variables. Particularly, we used the statistical software R version 3.0.0 with its function brunch, which is available in the R package caper (Comparative Analyses of Phylogenetics and Evolution in R) version 0.5. A phylogenetic relationship, which is required to perform phylogenetic comparative analysis, was generated based on a highly resolved tree of life [32] (see Figure S1 and Section 3.2 for details). As a result, we could not draw conclusions regarding the effect of oxygen on metabolic network expansion when considering phylogenetic relationships (Table 1a). This finding suggests that increased metabolic space due to oxygen utilization is taxonomically constrained (e.g., Figure 7 in Reference [26]). That is, a phylogenetic signal causes an association between the increased space and oxygen requirements. This means that an association between oxygen requirements and metabolic network expansion is not likely to exist.

However, note that this conclusion may be debatable, because phylogenetic comparative analyses have several practical and theoretical limitations [29]. Specifically, such analyses assume a simple evolutionary model, which deems random, Brownian-motion-like traits to be change on a phylogenetic tree with accurate branch lengths.

First, analysis of species, which can be investigated using phylogenetic comparative analyses, depends on the availability of phylogenetic trees. In this study, only ~20% (174) of organisms were investigated because a high-quality phylogenetic tree was used, although we collected metadata (*i.e*., oxygen requirements) for 930 organisms (see Section 3.1 for details). More comprehensive analysis is required to evaluate the association between oxygen and metabolic network expansion in greater detail.

More comprehensive phylogenetic trees (*i.e*., trees including more species) can be generated using 16S ribosomal RNA sequences or protein sequences; however, quality of the phylogenetic tree (e.g., branch lengths) affects the results of phylogenetic comparative analyses. To avoid this limitation, we used a highly resolved tree of life in this study. Moreover, if the assumption of Brownian motion is invalid, as when evolutionary trends have occurred, estimates may not be accurate.

### 2.2. Overestimated or Underestimated Differences of Chemical Properties between Oxic Metabolites and Anoxic Metabolites

Despite these limitations in data analysis, our findings cast doubt on whether oxygen availability increases chemical diversity in metabolic networks. Therefore, whether oxygen has led to the development of novel (*i.e*., oxic) metabolites with chemical properties different from anoxic metabolites [19] merely due to its presence remains unknown. Detailed examination is required as this conclusion has been derived through strongly biased evaluation, as described in Section 1.

To determine whether previous observations are overestimated, underestimated, or accurate, we investigated the degrees of differences in the chemical properties of oxic metabolites and anoxic metabolites in individual metabolic networks, and calculated the likelihood that the degree computed from the integral network was obtained from the set of degrees calculated from individual metabolic networks.

In this study, we considered the effect size of the Wilcoxon–Mann–Whitney (WMW) test, a nonparametric test, as an indication of the degree of differences between the chemical properties of oxic metabolites and anoxic metabolites. Note that the *p*-value is unsuitable for the degree of differences between two groups because sample size influences the *p*-value. Thus, we examined effect sizes. Effect sizes are normalized measures for statistical analyses that are not influenced by sample sizes. Effect size (*ES*) of the WMW test was obtained as follows:

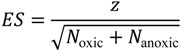
(1)
where *z* is the *z*-score calculated from the *p*-value obtained using the one-tailed WMW test and *N*_oxic_ and *N*_anoxic_ are the number of oxic metabolites and the number of anoxic metabolites, respectively. Particularly, *ES* > 0 or *ES* < 0 indicates that a chemical property’s median of oxic metabolites is larger or smaller, respectively, than that of anoxic metabolites examined in this study.

The likelihood that the effect size (*ES*_int_) of differences in chemical properties calculated from the integral networks was obtained from the set of effect sizes of the differences computed from individual metabolic networks was evaluated based on quantiles, which describe various subdivisions of a frequency distribution as they are distributed into equal proportions, as well as the evaluation value (*EV*) [33,34], which is defined as:

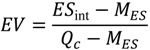
(2)
where *M_ES_* is the median (*i.e*., 50% quantile) of the effect sizes obtained from individual metabolic networks. *Q_c_* denotes the effect size at 2.5 or 97.5% quantiles, depending on whether *ES*_int_ is lower or higher than *M_ES_*.

Using *EV*, we evaluated whether an observed value (*i.e*., *ES*_int_) was significantly lower or higher than *M_ES_*. An *EV* larger than 1 indicates that *ES*_int_ is not within the most likely 95% of effect sizes from individual metabolic networks, suggesting an overestimation or underestimation of *ES*_int_ in individual metabolic networks.

Similarly, the *Z*-test can be used; however, this test assumes a normal distribution of variables. On the other hand, an evaluation using *EV* does not require assumptions regarding the distribution shape of the effect sizes obtained from individual metabolic networks; thus, it is more accurate than the *Z*-test.

We evaluated the differences (*i.e*., *E_int_*) in 84 chemical properties between oxic metabolites and anoxic metabolite, as were calculated in the previous study by Jiang *et al.* [19].

**Figure 2:**
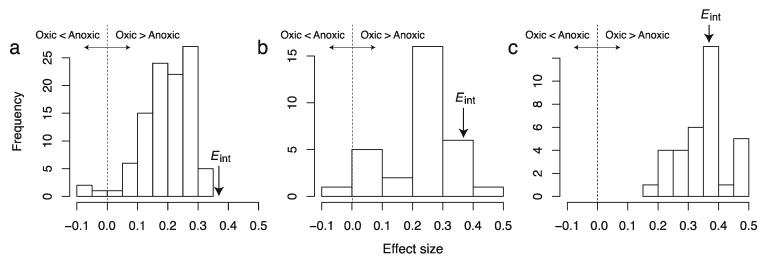
Distributions of effect sizes of the difference in AlogP98 between oxic and anoxic metabolites in individual metabolic networks of **(a)** aerobic bacteria, **(b)** unicellular eukaryotes, and **(c)** multicellular eukaryotes. *E_int_* is the effect size obtained from the integral network.

Jiang *et al.* found that hydrophobicity (e.g., measured as AlogP98, the logarithm of a water/octanol partition coefficient) of oxic metabolites is a discriminative chemical property (*i.e*., oxic metabolites are more hydrophobic than anoxic metabolites). As in the previous study, our analysis supported this conclusion when focusing on integral metabolic networks; however, we identified several overestimated and underestimated differences in the discriminative chemical properties of oxic metabolites, reported in the previous study, when considering the individual metabolic networks (Table 2) of non-redundant species belonging to the same domain to reduce phylogenetic effects as much as possible (see Section 3.1 and Tables S2 and S3 for details). In particular, the difference (*i.e*., *E_int_*) in hydrophobicity (e.g., AlogP98) between oxic metabolites and anoxic metabolites in the previous study were overestimated for aerobic unicellular organisms; however, this overestimation is not observed for multicellular eukaryotes (see also Figure 2). Similar conclusions can be derived from the context of molecular solubility and fraction of charged atoms distributed on the molecular surface area. Additionally, the toxicity of oxic metabolites was overestimated in aerobic unicellular organisms, although some descriptors reflecting hydrophobicity (e.g., SOL and FPSA) and toxicity (*i.e*., pLC50) were not highly overestimated in unicellular eukaryotes.

This result indicates that differences in hydrophobicity and toxicity are lower than previously thought, suggesting a lower impact of oxygen on increased chemical differences in this context. For example, alkaloids, which are a type of secondary metabolite in plant species and discriminative oxic metabolites, are strongly hydrophobic and toxic. It has been reported that other discriminative oxic metabolites are important for transmembrane export and import (steroids), signal transfer (steroids, diterpenoids, and polyphenols), defense against biotic factors (macrocyclic lactones in addition to alkaloids), and organism protection from oxidation (polyphenols) [19]; however, these metabolites should be observed characteristically in higher organisms such plants. When using the integral metabolic network to investigate differences between oxic and anoxic metabolites, these metabolites were weighted regardless of whether they are less conserved among species. In fact, differences in these chemical properties are lower in aerobic unicellular organisms because these organisms synthesize only small amounts of such metabolites. In contrast, neither overestimation nor underestimation was observed for multicellular eukaryotes, as expected.

However, observed overestimations and underestimations do not completely reject the contribution of oxygen to metabolic evolution, as differences in chemical properties are partially concluded. For example, 70% of aerobic bacteria and 80% of unicellular eukaryotes showed significant difference in AlogP98 between oxic and anoxic metabolites (*p*-value < 0.01, WMW test). Additionally, the rigidity of rotatable bonds (*i.e*., RotBonds in Table 2), another discriminative chemical property of oxic metabolites examined in the previous study [19], was not overestimated for individual metabolic networks. This is because oxidoreductases, which are enriched in oxic enzymes [19], contribute to increased rigidity because they convert single bonds and double bonds between two atoms.

**Table 2:** Likelihood (evaluation value *EV*) that the effect size (*E_int_*) of differences in chemical properties between oxic metabolites and anoxic metabolites calculated from the integral networks was obtained from the set of effect sizes of the differences computed from individual metabolic networks. A positive and negative effect size indicates that the value for a chemical property of oxic metabolites was larger and smaller, respectively, than that of anoxic metabolites. An *EV* value of larger than 1 indicates overestimation or underestimation of (*E_int_*) in an individual network. *P* corresponds to the negative logarithmic *p*-value (*i.e*., –log_10_(*p*-value)) obtained from the Wilcoxon–Mann–Whitney (WMW) test. *M_ES_* and *M_P_* denote the median of *P*s and *ES*s in individual networks. 6 representative chemical properties between oxic metabolites and anoxic metabolites in a previous study [19] are shown. The descriptors are as follows: the logarithm of partition coefficient, atom-type value, using latest parameters (AlogP98), molecular solubility (SOL), ratio of atomic charge weighted partial negative surface area on total molecular surface area (FNSA), ratio of atomic charge weighted partial positive surface area on total molecular surface area (FPSA), negative log of lethal concentration 50% (pLC50), and rotatable bond count (RotBonds).

Descriptor	Integral		Aerobic bacteria			Unicellular eukaryotes			Multicellular eukaryotes		
	*E*_int_	*P*	*M_ES_*	*M_P_*	*EV*	*M_ES_*	*M_P_*	*EV*	*M_ES_*	*M_P_*	*EV*
AlogP98	0.37	34.9	0.21	3.76	1.56	0.26	5.72	1.27	0.36	11.2	0.08
SOL	−0.34	30.1	−0.17	2.58	2.03	−0.29	6.92	0.67	−0.33	10.1	0.07
FNSA	0.32	26.4	0.13	1.78	1.08	0.16	2.43	1.00	0.26	7.06	0.30
FPSA	−0.23	13.8	−0.06	0.58	1.90	−0.14	2.01	0.60	−0.22	4.36	0.10
pLC50	0.27	19.7	0.09	1.00	1.70	0.21	3.93	0.86	0.27	6.75	0.01
RotBonds	−0.23	13.8	−0.27	5.78	0.38	−0.27	4.59	0.24	−0.28	7.60	0.49

### 2.3. Does Oxygen Contribute to Metabolic Evolution?

The observation that network expansion was not conclusive when considering a phylogenetic relationship, and that overestimations or underestimations of the difference in chemical properties between oxic metabolites and anoxic metabolites were observed in aerobic unicellular organisms (particularly in aerobic bacteria) but not in multicellular organisms, suggests a limited association between oxygen requirements and chemical differences. Rather, phylogenetic signals and multicellularity (*i.e*., cell-cell communication) increase chemical diversity in metabolic networks.

Many studies state that oxygen accelerates evolution, such the emergence of multicellularity (e.g., reviewed in [11,12]), because most multicellular organisms showing great diversity (in the context of metabolites in this study) are obligately aerobic. This result suggests an association between oxygen and evolution; however, obligately aerobic unicellular organisms are less complex. For example, differences in chemical properties between oxic and anoxic metabolites are less significant than previously thought in obligately aerobic bacteria (Table 2 and Figure 2). Thus, its remains possible that the contribution of oxygen to metabolic evolution is an artifact due to strongly biased observations of (aerobic) multicellular organisms, as discussed in Section 2.2.

To more effectively identify the presence of an association between oxygen and metabolic evolution, investigation of anaerobic multicellular organisms is required. For example, Danovaro *et al.* [35] demonstrated that a representative from the animal phylum Loricifera lives in a deep, permanently anaerobic, and hypersaline water basin in the Mediterranean Sea. This organism may be useful in investigating whether oxygen contributes to molecular diversity, although its genomic and metabolic data are not available. If oxygen accelerates the increase in molecular diversity, then such an increase in diversity should not be observed for anaerobic multicellular organisms.

An opposite hypothesis is that the contribution of oxygen to metabolic evolution is limited and can be examined by investigating anaerobic multicellular organisms. Our hypothesis assumes that an association between oxygen and metabolic evolution is a side effect of the relationship between oxygen requirements and multicellularity. In particular, the acquirement of multicellularity may be a strategy for protecting against oxidative stress. Oxygen is useful for energy production; however, it also plays a role as a biomolecular denaturant. Unicellular organisms are quite susceptible to oxidative stress, but they can avoid such stress by aggregating to reduce their exposed surface areas. In this case, cell-cell communication is required. Thus, unicellular organisms acquired more hydrophobic compounds for signal transfer (*i.e*., communications between membranes), and finally evolved into multicellular organisms. This hypothesis may be supported that fact that the difference in chemical properties between oxic metabolites and anoxic metabolites are significant in multicellular organisms, but not in unicellular organisms (aerobic bacteria, in particular) (Table 2). This indicates that oxygen is just one possible reason for the emergence of multicellularity (*i.e*., cell-cell communication), suggesting that increased chemical diversity was influenced by cell-cell communication rather than oxygen. Therefore, if our hypothesis is more fitting, anaerobic multicellular organisms show increased chemical diversity, such as network expansion and overestimated differences in the chemical properties between oxic and anoxic metabolites, which contradicts the classical hypothesis.

Our conclusion that oxygen has a weak effect on evolution is limited to the context of chemical diversity in metabolic networks. As described in Section 1, many previous studies have provided evidence that oxygen contributes to species and morphological diversity in higher organisms such as fish and insects. Such macroscopic evolution after the emergence of multicellular organisms may be explained in a different context.

Our analysis (in Section 2.1 and Section 2.2) has general limitations, as do many other works on metabolic network analyses, including limited knowledge of metabolic reactions (*i.e*., missing links), reconstruction of metabolic networks based on genomic information. That is, it remains possible that our conclusion is affected by the difference in the percentage of functionally-unknown proteins in a species genome between aerobes and anaerobes. Thereby, we evaluated the fraction of functionally-unknown proteins in genome for each species (see Section 3.5).

When using the dataset (see Table S1), for example, we cannot conclude any difference in the fraction of functionally-unknown proteins among aerobes, facultative aerobes, and anaerobes (Figure S2). (*p* = 0.33 using the Kruskal-Wallis test, whose alternative hypothesis is that at least one group is different from the other groups). Thus, the difference in the fraction of functionally-unknown proteins might not seriously affect our conclusions. 

Nevertheless, metabolic networks have not been fully understood; thus, there is a need for a more careful examination in data analysis in the future. For example, enzyme promiscuity [36], which implies that enzymes can catalyze multiple reactions, act on more than one substrate, or exert a range of suppressions [37], in which an enzymatic function is suppressed by over-expressing enzymes showing originally different functions, suggests the existence of many hidden metabolic reactions. Consideration of these hidden metabolic reactions is important for designing metabolic pathways and understanding metabolic evolution.

Although data analysis has these limitations, our finding encourages a more careful examination of an association between oxygen and evolution in both macroscopic and microscopic contexts.

## 3. Materials and Methods

### 3.1. Species Selection

We collected the data on organisms’ phenotypes (*i.e*., oxygen requirement, growth temperature, symbiosis, and multicellularity), are available in the supplemental materials in the previous studies [20,21,23,24], and selected 930 organisms, including 798 bacteria, 45 archaea, and 87 eukaryotes, for which data on metabolic networks were available in the KEGG (Kyoto Encyclopedia of Genes and Genomes) database [38], which are widely used and contain the metabolic pathways of many living organisms, including 201 eukaryotes, 2,469 bacteria, and 160 archaea (as of 17 September 2013).

The phylogenetic comparative method (Section 2.1) is widely used when comparing between species traits (increase rates and oxygen requirements, in this study) using phylogenetic relationship among organisms because these traits are all related through their hierarchical phylogenetic history although traditional interspecific comparative methods assume the statistical independence of observations, and assumes that a Brownian motion (random walk in continuous time) model of character evolution (see [26,27,28,29] for details), in which branch lengths would necessarily be proportional to divergence times; thus, it requires a phylogenetic tree. We selected 174 organisms (Table S1) from 930 organisms, including 133 bacteria, 18 archaea, and 23 eukaryotes, according to organisms available in the highly resolved tree of life [32] (see Section 3.2 for details).

The analysis in Section 2.2 cannot consider a phylogenetic relationship among organisms. Thus, to prevent redundancies and reduce phylogenetic signals, we extracted one species from a genus according to the year in which the species genome was first completely determined. The year in which the complete genome was published is based on the KEGG database (e.g., see [39]) Moreover, free-living and mesophilic organisms were selected to remove the effect of differences in species habitats and growth temperatures. Finally, we obtained 105 strictly aerobic bacteria (including no facultative aerobes) (Table S2) and 67 eukaryotes (Table S3), including 33 unicellular organisms and 34 multicellular organisms. We omitted archaea from this analysis because only a few samples were obtained after data handling.

### 3.2. Phylogenetic Tree

A highly resolved tree of life [32], which included 151 bacteria, 18 archaea, and 23 eukaryotes, was used for comparative phylogenetic analysis. Using iTOL (interactive Tree Of Life) editor [40], we constructed the phylogenetic tree, investigated in this study, by removing the leaves (organisms) whose oxygen requirement and multicellularity were available in the dataset described in Section 3.1, from the original tree [32] (see Section 3.1 for the composition of each domain). However, since only ~9% (17/174) bacteria (see Table S4) was removed from the original tree, the phylogenetic tree, investigated in this study, is almost similar with the original tree.

### 3.3. Data on Oxic and Anoxic Metabolism

According to a database [17] (prelude.bu.edu/O2/networks.html), in which identified oxic reactions and anoxic reactions are listed based on Enzyme Commission (EC) numbers and metabolic reaction notations (*i.e*., reaction (R) numbers such as R00010) in the KEGG database [38], we identified 1,627 oxic enzymes and 1,225 anoxic enzymes. Based on R numbers, we identified 1,679 oxic metabolites 1,236 anoxic metabolites using the file reaction_mapformula.lst, describing only the primary metabolites in each reaction (as in the KEGG pathway diagrams) and not the co-factors (e.g., water and ATP). This file was downloaded from the KEGG FTP site [41] on 23 May 2011. Note that the KEGG FTP site was available only to paid subscribers as of 1 July 2011. Since the use of such data may be desirable to ensure reproducibility, our dataset on metabolic networks is available upon request.

### 3.4. Chemical Properties

We identified metabolites whose chemical properties were made available in the previous study [19] according to the Compound numbers (e.g., C00022: pyruvate) in the KEGG database. These metabolites have clearly defined structures (*i.e*., without R group or polymeric form). Oxic intermediate metabolites were omitted (*i.e*., metabolites leaded from the “augmented reaction”, defined as in [17]) in order to perform data analysis under the same conditions as used in the previous study [19]. Finally, we investigated 651 oxic metabolites and 1,093 anoxic metabolites.

### 3.5. Functional Annotation

We downloaded the data on functional annotations of proteins in the organisms, investigated in this study, from the KEGG FTP site [41] on 26 May 2013. As mentioned in Section 3.3, the KEGG FTP site was available only to paid subscribers; thus, the dataset is available upon request.

According to the data on functional annotations, we define the functionally-unknown proteins as proteins whose annotations include at least one of the following words: “hypothetical”, “predicted”, “putative”, “unknown”, “-related”, “-family”, “-like”, and “probable”. Finally, we calculated the fraction of these proteins in genome for each organism (*i.e*., the number of functionally-unknown proteins divided by the total number of proteins encoded in genome).

## 4. Conclusions

In this study, we came to two major conclusions. First, metabolic network expansion due to an increased use of oxygen could not be clearly established when considering phylogenetic relationships. Second, differences in chemical properties between oxic metabolites and anoxic metabolites are less significant than previously hypothesized. These results suggest a limited influence of oxygen on metabolic evolution, although the hypothesis that oxygen accelerates metabolic evolution is widely accepted in biology. However, our findings do not contradict such a traditional hypothesis; rather, they require more careful examination using more comprehensive analysis (e.g., including anaerobic multicellular organisms). This study may provide new and different insights into the association between oxygen availability and metabolic evolution, despite several limitations in data analysis, and may shed light on the effect of oxygen on biological evolution.

## Supplementary Materials

Supplementary materials can be accessed at: http://www.mdpi.com/2218-1989/3/4/979.

Supplementary File 1 Supplementary (ZIP, 849 KB)
