# Supplementary material for: Limited Influence of Oxygen on the Evolution of Chemical Diversity in Metabolic Networks
Source: Metabolites. 2013 Oct 16;3(4):979–92. doi: 10.3390/metabo3040979 (PMC3937826; doi:10.3390/metabo3040979)
Supplement: Supplementary File 1 — Supplementary (ZIP, 849 KB) [file metabolites-03-00979-s001.zip › metabolites-03-00979-supplementary/Supplementary_Material_1.docx]

**Supplementary Material**

**Figure S1.** The phylogenetic tree used in the phylogenetic comparative analysis in Section 2.1 (see also Table S1). The aerobes, facultative aerobes, and anaerobes are shown in magenta, cyan, and black, respectively.

**Figure S2.** The ratio of functionally-unknown proteins (see Section 3.5 for details) on all proteins encoded in genome with respect to oxygen requirement (see Table S1 for the investigated organisms).
